# Prognostic impact of tumor location in colon cancer: the Monitoring of Cancer Incidence in Japan (MCIJ) project

**DOI:** 10.1186/s12885-019-5644-y

**Published:** 2019-05-09

**Authors:** Hiroko Nakagawa-Senda, Megumi Hori, Tomohiro Matsuda, Hidemi Ito

**Affiliations:** 1 Department of Public Health, Nagoya City University Graduate School of Medical Science, Nagoya, Japan; 2 Division of Cancer Statistics Integration, Center for Cancer Control and Information Services, National Cancer Center, Tokyo, Japan; 3 Division of Surveillance, Center for Cancer Control and Information Service, National Cancer Center, Tokyo, Japan; 4 Division of Cancer Information and Control, Department of Preventive Medicine, Aichi Cancer Center Research Institute, 1-1 Kanokoden Chikusa-ku, Nagoya, Aichi 464-8681 Japan; 5 Department of Epidemiology, Nagoya University Graduate School of Medicine, Nagoya, Japan

**Keywords:** Population-based cancer registries, Colorectal cancer, Net survival, Anatomical subsite

## Abstract

**Background:**

Colorectal cancer (CRC) is globally one of the most common cancers. Although studies have found a significant prognostic impact of cancer location for right-sided colon cancers compared with those of the left-side, evidence is lacking in a Japanese population. Therefore, we investigated 5-year net survival in colon cancer by tumor site in a Japanese population.

**Methods:**

Diagnoses obtained between 2006 and 2008 in 21 population-based cancer registries from the Monitoring of Cancer Incidence in Japan (MCIJ) project were used. Colon cancer patients were categorized as having right-sided (C18.0–18.4) or left-sided colon cancer (C18.5-C18.7). We calculated the 5-year net survival for subjects diagnosed from 2006 until 2008 by anatomical subsite according to sex, age groups, tumor stage at diagnosis. We applied the excess mortality model to calculate excess hazard ratios (EHRs) and 95% confidential intervals (CIs) with and without adjustment for age, sex and cancer stages to evaluate the effect of location of colon cancer.

**Results:**

This study analyzed a total of 62,350 colon cancer subjects. Five-year net survivals for subjects with left- and right-sided colon cancer were 74.0% (95% CI, 73.4–74.7%) and 70.4% (95% CI, 69.7–71.0%), respectively. Compared with left-sided colon cancers, the EHR for right-sided colon cancers was 1.20 (95% CI, 1.16–1.25) after adjustment for age, sex and stage.

**Conclusion:**

Our study found that the net survival for right-sided colon cancer was significantly lower than that for left-sided colon cancer. The anatomical site of cancer in the colon might be an important stratification factor in future studies of colon cancer.

**Electronic supplementary material:**

The online version of this article (10.1186/s12885-019-5644-y) contains supplementary material, which is available to authorized users.

## Background

Colorectal cancer (CRC) is globally one of the most common cancers [1]. In 2012, the estimated incidence was 1,360,000 new patients and 694,000 deaths worldwide, accounting for 8.5% of total deaths [1]. The incidence and mortality of CRC have increased dramatically during the last several decades in a Japanese population [1–3]. In 2017, CRC was the most common cause of cancer death in women and the third-most common in men, with the 50,700 patients who died due to CRC accounting for 3.7% of total deaths in Japan [4].

The differentiation of colon cancer by anatomical subsite has received substantial attention over the past decade. The clinical and biological characteristics of CRC are different according to the anatomical subsites of the colon tumor [5, 6]. Recent studies have revealed that the frequency or incidence of right-sided colon cancer has increased during the past decade while that of left-sided colon or rectal cancer has remained stable or decreased [3, 7, 8]. Epidemiological studies have indicated that the impact of risk or protective factors on CRC might differ by colorectal anatomical subsites [9–13]. A recent systematic review noted that many studies have identified differences by anatomical subsite with regard to epidemiology, clinical presentation, pathology and genetic mutations [5]. These findings have in turn led to suggestions that the location of colon cancer may influence prognosis.

A number of epidemiological studies have reported the association between prognosis and cancer location in the colon. In 2016, a meta-analysis of 66 studies suggested that there was a significant prognostic impact of the tumor site, with an 18% increase in mortality risk for cancers arising from the right side [14]. Although most of these studies demonstrated poorer survival in right- than left-sided colon cancer [14–19], others are inconsistent [20, 21]. Contrary to these other studies, however, one recent population-based analysis suggested that the prognosis of left-sided colon cancer is worse than that of right-sided colon cancer [20]. In Japan, only a few studies have reported associations between cancer location in the colon and prognosis [17, 18, 21–23] namely poorer survival in right- than left-sided tumor [17, 18], better survival in right- than left-sided tumor [21] or no difference in survival between them [22, 23]. Thus, evidence to prove that the prognosis of colon cancer differs by side in a Japanese population is lacking.

Here, we aimed to investigate the net survival of patients with right- and left- sided colon cancers using data from population-based cancer registries in a Japanese population.

## Methods

Using population-based cancer registries data from the Monitoring of Cancer Incidence in Japan (MCIJ) project, we analyzed colon cancer cases (ICD-10: C18.0–18.7) diagnosed from 2006 until 2008 in 21 population-based cancer registries (Aichi, Chiba, Ehime, Fukui, Fukushima, Gunma, Hiroshima, Ibaraki, Kanagawa, Kumamoto, Miyagi, Nagasaki, Niigata, Osaka, Okayama, Shiga, Shimane, Tochigi, Tottori, Yamagata and Yamanashi) in Japan. Cases were selected according to Japanese standards with regard to (i) proportion of cases reported by death certificate only (DCO%: death certificate only) of less than 25%, (ii) proportion of cases first notified through death certificate (DCN%: death certificate notification) of less than 30%, (iii) mortality to incidence ratio (M/I) of less than 0.67 [24], and (iv) percentage of lost to follow-up of < 5% or adopted linkage to a death certificate database to confirm the vital status of patients. We included those patients diagnosed in 2006–2008 and followed through Dec 31, 2013. Japanese population-based cancer registries start to follow patients at the date of diagnosis and do not register the date of operation or starting treatment. We excluded data from cases that were registered by death certificate only, were secondary multiple cancers, were in situ cases, and those in patients aged > 100 years. We also excluded data from cases that were registered by death certificate notification. The study included colon cancer cases (ICD-10: C18.0–18.7), cecum, C18.0; appendix, C18.1; ascending colon, C18.2; hepatic flexure of the colon, C18.3; transverse colon, C18.4; splenic flexure of the colon, C18.5; descending colon, and C18.6; and sigmoid colon, C18.7. Overlapping lesions of colon (C18.8) and those not otherwise specified (C18.9) were excluded. Colon cancer patients were further categorized into two groups, those with right-sided colon cancer (C18.0–18.4; cecum, appendix vermiformis, ascending colon, hepatic flexure of colon and transversal colon) and left-sided colon cancer (C18.5-C18.7; splenic flexure of colon, descending colon and sigmoid colon). With regard to the extent of disease, patients were categorized into the three disease stages of localized, regional and distant groups. Extent of disease was available in the Japanese population-based cancer registries. The Japanese staging system, extent of disease, was based on the Surveillance, Epidemiology, and End Results (SEER) staging criteria [25]. Extent of disease was unknown for 14.0% of subjects.

### Statistical analysis

The frequency of related variables of patients by cancer locations was compared using the two sample t-test for continuous variables and the χ^2^ test for categorical variables. We calculated 5-year net survival for colon cancer patients diagnosed from 2006 until 2008 by anatomical subsite according to sex, age group (< 40, 40–54, 55–69, ≥70), extent of disease at diagnosis (localized, regional or distant stages). Net survival is regarded as the survival that would be observed in the hypothetical situation that the only possible cause of death was cancer [26]. Net survival is calculated by following two methods: relative survival and cause-specific survival. The population-based cancer registries usually use relative survival to give estimates net survival [27]. We used the recently introduced Pohar Perme estimator [28] of net survival implemented with the program *stns* in Stata version 14.1. The complete national population life-tables by single year of age, sex and calendar year were used to derive the expected mortality rates. To assess the impact of anatomical location of the colon cancer on survival, the excess mortality model, a multivariate regression approach which adopts the flexible parametric model [29, 30] implemented with the *stpm2* function in Stata version 14.1 was used. We applied the excess mortality model to calculate excess hazard ratios (EHRs) and 95% confidential intervals (CIs) with and without adjustment for age, sex and cancer stages to assess the effect of the location of colon cancer. Cases in which the tumor stage was unknown were excluded when the excess mortality model was performed to adjust for tumor stage. The differences in survival rate with location of colon cancer between sex, age groups or tumor stages were statistically tested by including an interaction term into the excess mortality model. A two-sided *P*-value of < 0.05 was considered statistically significant. All statistical analyses were performed using Stata v. 14.1 (STATA Corporation, College Station, TX).

## Results

### Characteristics of subjects

Information on a total of 62,350 subjects diagnosed with colon cancer from 2006 until 2008 was analyzed, of whom 32,005 (51.4%) had right-sided disease and 30,345 (48.6%) had left-sided. The distribution of demographic variables among the subjects are shown in Table 1. Of these 62,350 patients, 53.8% were 70 years of age or older and 53.4% were male. With regard to tumor stages, most patients were diagnosed with localized disease (41.1%), followed by regional (27.7%), distant (17.3%) and stage unknown (14.0%). There were differences among tumor locations in age, sex and stage. Patients with right-sided cancer were significantly older (mean age 71.2 ± 11.5 vs 67.9 ± 11.4 years old), more likely to be female (52.3% vs 40.7%), and had a higher percentage of distant stage disease (18.0% vs 16.5%) (*p* < 0.001), compared to those with left-sided disease.

**Table 1:**
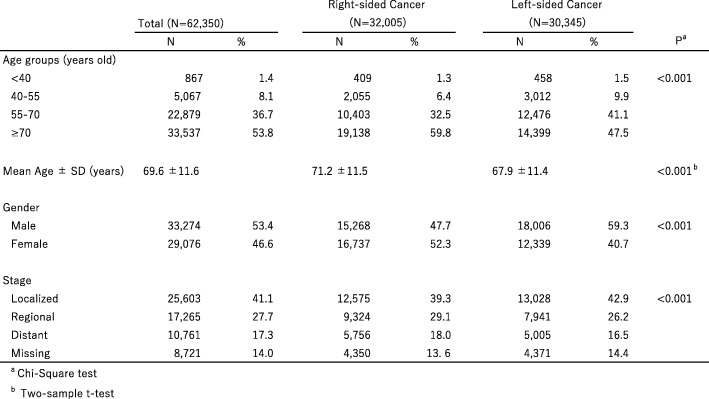
Patient characteristics

### Survival analysis

**Fig. 1:**
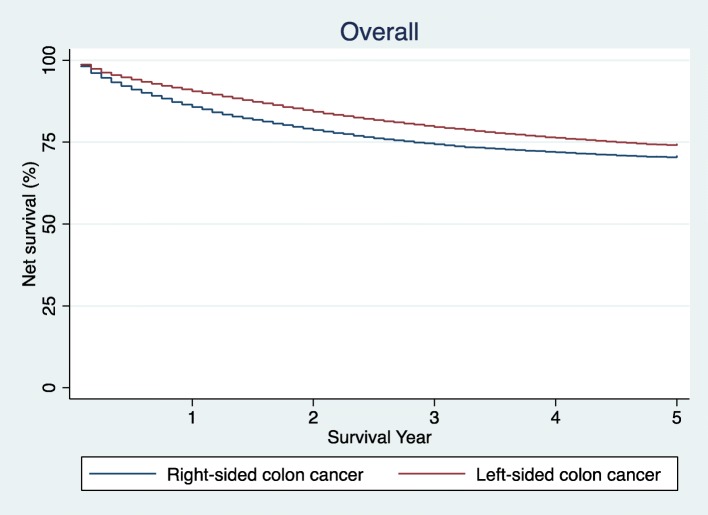
5-year net survival for patients with right- and left-sided colon cancer. Net survival rates up to 5 years were shown in blue for right-sided colon cancer and in red for left-sided colon cancer

Table 2 shows the 5-year net survival and estimated excess hazard ratios for colon cancer by sex, age group, disease stage and anatomic location. The 5-year net survival was lower in females than in males. Further, it decreased with increasing age after adjustment for sex and stage, and decreased with advancing stage after adjustment for sex and age. The 5-year net survival estimates for colon cancer by anatomical subsite are shown in Fig. 1, at 74.0% (95% CI, 73.4–74.7%) for subjects with left-sided colon cancer and 70.4% (95% CI, 69.7–71.0%) for right-sided disease. Compared with left-sided colon cancers, EHR for right-sided cancers was 1.20 (95% CI, 1.16–1.25) after adjustment for age, sex and tumor stage (Table 2).

**Table 2:**
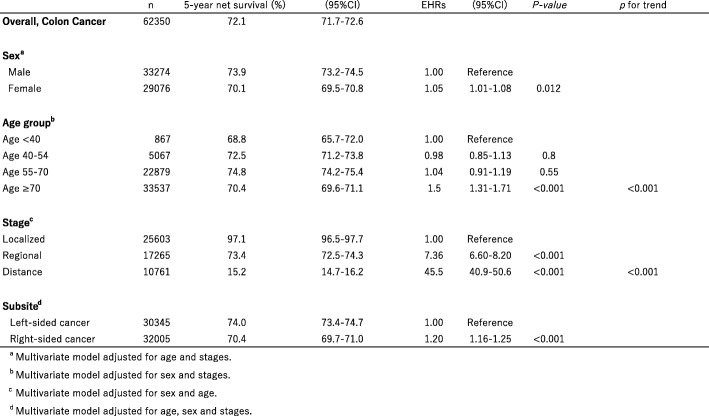
5-year net survival (%) and estimated excess hazard ratios for colon cancer by sex, age, group, stage and subsite, Japan, 2006-2008

The 5-year net survival for subjects with left- and right-sided colon cancer by sex, age group and tumor stage are also shown in Table 3. Five-year net survival for subjects with left- and right-sided disease were 74.5% (95% CI, 73.6–75.3%) and 73.2% (95% CI, 72.2–74.2%) for males, and 73.4% (95% CI, 72.4–74.3%) and 67.8% (95% CI, 66.9–68.7%) for females, respectively. Compared with left-sided disease, EHRs for right-sided disease were 1.19 (95% CI, 1.14–1.26) for males and 1.19 (95% CI, 1.13–1.26) for females after adjustment for age and stage. No heterogeneity was found between sexes (*P* = 0.39). With regard to age groups, 5-year net survival was lower for right-sided than left-sided disease in all age groups (Additional file 1: Figure S1A-D). Compared with left-sided cancers, EHRs for right-sided cancers were 1.09 (95% CI, 0.84–1.43) for age less than 40 years, 1.32 (95% CI, 1.18–1.48) for age 40–54 years, 1.15 (95% CI, 1.08–1.21) for age 55–70 years, and 1.26 (95% CI, 1.19–1.33) for age ≥ 70 years, respectively, after adjustment for sex and stage. Statistically marginal heterogeneity was found among these age groups (*P* = 0.05). Survival differences by anatomic subsite were observed for those aged 40 or over, whereas significant difference was not observed for those aged less than 40 years. By stage, 5-year net survival for right-sided disease was also lower than that for left-sided disease in regional and distant disease but higher in localized disease (Additional file 2: Figure S2A-C). EHRs for right-sided colon cancers, compared with left-sided, were 0.74 (95% CI, 0.60–0.90) for stage localized, 1.25 (95% CI,1.17–1.34) for stage regional, and 1.20 (95% CI, 1.15–1.25) for stage distant, respectively, after adjustment for sex and age. Heterogeneity was marginally significant among stages (*P* = 0.07).

**Table 3:**
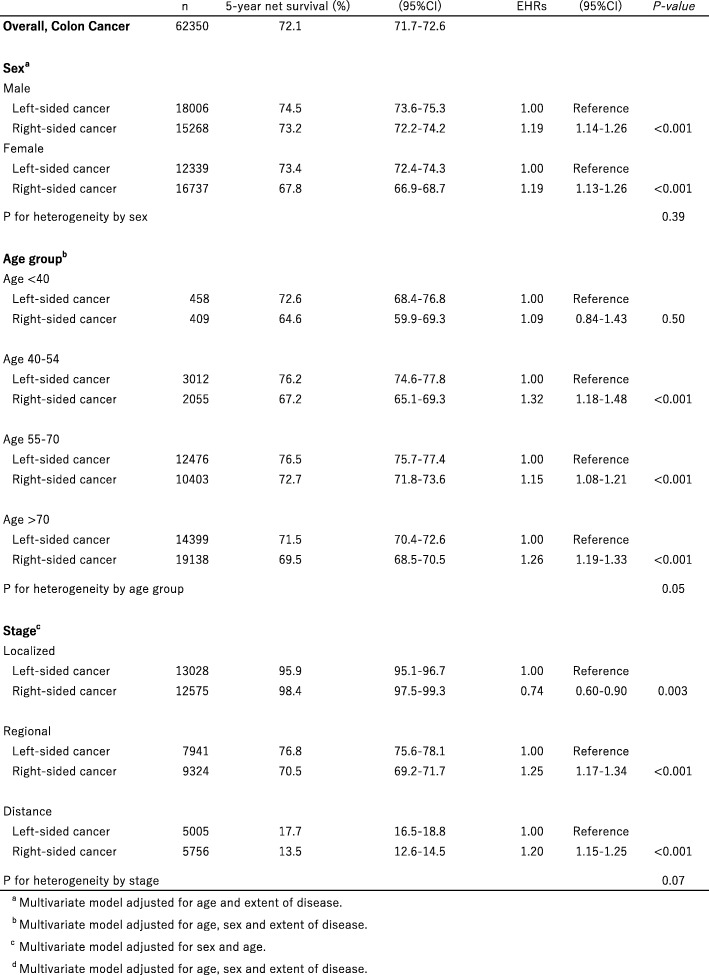
5-year net survival (%) and estimated excess hazard ratios for colon cancer by subsite according to sex, age group and stage subsites, Japan, 2006-2008

The results by age group and stage were consistent between the sexes when stratified by sex (Additional file 3: Table S1 and Additional file 4: Table S2).

## Discussion

In this study, we showed that survival of subjects with right-sided colon cancer was lower than that of subjects with left-sided disease with assessment for adjusted EHRs. On stratification by age group, survival for right-sided disease was lower than that for left-sided disease in those aged 40 years or over, with assessment for adjusted EHRs. On stratification by tumor stage, survival for right-sided colon cancer was significantly lower than for left sided disease in regional and distant stage disease, but higher in localized disease. To our understanding, this is the first study to evaluate population-based cancer registry data using the unbiased Pohar Perme estimator of net survival to assess the effect of anatomical subsite on survival of colon cancer patients. Among previous studies on the association between the location and prognosis, a meta-analysis study reported that patients with right-sided colon cancers had an 18% increase in mortality risk and that this was independent of stage [14], which is similar to our result. Analyses using SEER data found that right-sided colon cancers were associated with a 4% increased risk of death compared with cancers of left-sided cases after adjustment for confounders [15]. However, a more recent analysis using the SEER database provided evidence that while right-sided cancer patients were associated with worse overall survival than left-sided disease patients, this relationship was reversed after propensity score matching, rendering the prognosis of cancers with right-sided better overall [20]. The authors speculated that differences among confounders that could not be adjusted for in multivariate regression analysis caused this reversal of results.

Differences in distribution by stage and age have an important effect on survival rate [20, 31]. Patients with a more advanced stage and older age at diagnosis had a greater increase in mortality risk [20, 31]. Compared with those aged < 40 years, hazard ratios for overall mortality were 1.20 (95% CI,1.12–1.28) for age 50–64 years, 2.30 (95% CI, 2.15–2.45) for age 65–79 years, and 5.10 (95% CI, 4.77–5.47) for age ≥ 80 years, respectively [20]. For this reason, we estimated the difference in survival by anatomical subsite with adjustment for stage and age groups. We confirmed that anatomical subsite was an independent prognostic factor for patients with colon cancer. Subsites within the colorectum are derived from distinct embryonic origins [5]. The survival differences between right- and left-sided colon cancer may have resulted from differences between subsites in epidemiology, genetic mutations, pathology and clinical features [5].

Epidemiological analyses of data from Japanese cancer registries and SEER have shown that incidence rate trends for proximal colon cancer differ from those of distal disease [3, 8]. Epidemiological studies found evidence that the impact of risk factors for CRC, including low physical activity and meat consumption, and protective factors, including coffee intake and aspirin use, differ by anatomical subsite [9–13]. Differences in gene expression between cancers in right- and left-sided colon have been evaluated: while right-sided cancers are characterized by *BRAF* mutation, high microsatellite instability (MSI), and CpG island methylation [32–34], left-sided cancers frequently have p53 and *KRAS* mutation [35]. BRAF mutations are a part of the RAS-RAF-MAP2K (MEK)-MAPK signaling pathway. *BRAF* mutation cancers were associated with worse overall survival than wild-type cancers [32, 33, 36]. CpG island methylation-positive tumors showed significantly worse outcomes than those with negative tumors [34]. These findings are consistent with our result. Patients with MSI-positive cancers nevertheless show better survival than those with cancers exhibiting microsatellite stability (MSS) [37], which is inconsistent with our results. Only a few studies have evaluated the combined impact of CpG island methylation, BRAF mutation status and MSI status on survival for colon cancer [38]. The mechanism of the difference in survival by location of colon cancer warrants further study. We found that survival was significantly lower for right-sided disease than for left-sided disease in patients aged ≥40. Although we observed no significant difference among those aged less than 40 years, and that the association was not statistically significant, the point estimates for the effect measures showed the same direction, with EHRs of more than 1.0. The lower survival in right-sided colon cancer might be robust in all age groups.

Our findings suggest that the anatomical site of colon cancer might be a crucial factor in establishing prognosis, particularly in advanced-stage disease. Prognosis for right-sided colon cancers was worse in stage III or IV according to the American Joint Commission on Cancer (AJCC), but did not differ or was better in AJCC stage I or II [15, 16, 19], which is consistent with our results. In Japan, while a few hospital-based studies have appeared [17, 18, 21–23], we are unaware of any study which has used population-based data to examine the association between the location and prognosis of colon cancer. The prognosis for cancer in the right-sided of the colon is worse than for disease in the leftside in stage III [17] and IV colon cancer [18], but better in stage I [21]. Our present results are consistent with these findings. In contrast, two other studies reported that no difference was observed in prognosis between cancers in right-sided and left-sided colon [22, 23].

The reasons for this inconsistent association between survival and anatomical cancer location by disease stage is not clear and warrants further study. One possibility might relate to cancer biology such as MSI status. MSI-positive tumors, which are mainly seen in right-sided colon, have been associated with improved prognosis [39]. MSI has a favorable stage profile. This inconsistent association might owe to the difference in the percentage of MSI-positive colon cancers according to stage [16]: MSI positivity in right-sided colon cancers was most frequent in stage II cancers, and less frequent in the order of stage III and stage IV disease [40]. Because MSI is predominantly seen in colon cancers of the right side, we assume that earlier stage right-sided disease could have a higher frequency of MSI positivity than left-sided disease at the same stage, but that this difference diminishes with increasing stage. In contrast, CpG island methylation and BRAF mutation do not appear to have a favorable stage profile. This may cause the inconsistent association seen between survival and tumor location by stage. To our understanding, however, no study has yet investigated the percentage of MSI-positive tumors according to cancer location and stage, or the influence of CpG island methylation, MSI status and BRAF mutation status in combination on survival by stage and subsite for colon cancer. The reason for the inconsistency in survival therefore remains unclear, and warrant further study.

This study has several strengths. First, we examined survival in colon cancers by anatomical subsites using data from a large population-based cancer registry in Japan. The population of the 21 prefectures was 60,117,000 in 2006, accounting for 47.1% of the total Japanese population. The use of population-based data allowed us to evaluate the actual prognostic effect of anatomical subsites in people with heterogeneous backgrounds in the general population. Second, we calculated net survival with the newly introduced Pohar Perme estimator to show unbiased net survival. This estimator provides findings that are unaffected by deaths not related to this cancer, and is therefore the preferred standard for estimating net survival [41]. In addition, we applied the recently introduced flexible parametric model to evaluate the impacts of anatomical subsites of colon cancer in survival. Although Poisson regression models are popular, the recently developed flexible parametric model, first proposed by Royston and Parmar [29] and applied to relative survival model by Nelson et al. [30], has a number of advantages. First, it offers smooth estimates of excess mortality rates and relative survival on the log cumulative excess hazard scale through the use of restricted cubic splines. Other advantages include the ability to model time on a continuous scale, the provision of hazard functions and survival in an analytical manner, and the elimination of need for the use of split-time data [30].

This study also has several limitations. First, information on family history, performance status and comorbidities are not available in the MCIJ dataset. These factors might play a role in patient outcome, albeit to an unclear extent. Second, we can not obtain information on *BRAF* mutation, MSI, CpG island methylation and chemotherapeutic treatment from the MCIJ data. Since the middle of the 2000’s, oxaliplatin with a fluoropyrimidine has been standard adjuvant chemotherapy for patients with stage III colon cancer, and is suggested to improve overall survival [42]. Information on adjuvant chemotherapy in the colon cancer patients with stage III also can not be ascertained from the MCIJ data, and we were unable to adjust for the use of adjuvant chemotherapy in this study. In addition, only extent of disease, and not specific stage groupings, was available in the Japanese population-based cancer registries. Furthermore, 14% of the subjects were diagnosed with stage unknown. However, because the proportion of stage unknown patients did not differ among the anatomical subsites, we believe that the effects of this stage unknown status are likely small.

Finally, the Japanese population-based cancer registries had issues with quality during the study period, and failed to meet data quality for international standards for the proportion of death-certificate-only. When hospitals do not report cancer patients and the patients survive, the assumption will be biased and survival rates might be underestimated. In addition, inclusion of death certificate notification cases in cases of death will also cause bias, and survival might be underestimated. For these reasons, we excluded data for cases that were registered by death certificate notification. Enactment of the new Promotion of Cancer Registries Law in 2016 will bring about an improvement in the data quality.

## Conclusions

This study revealed the net survival for colon cancer by anatomical subsite using large population-based cancer registries data in a Japanese population. Net survival for right-sided colon cancer was significantly lower than that for left-sided disease. This finding suggests that right-sided colon cancer might be biologically more aggressive than left-sided colon cancer. Determining or comparing the biological profiles of colon cancers between right- and left-sided, including genetic changes, will elucidate the underlying mechanism. Anatomical site of cancer in the colon might suggest crucial stratification factors for future studies of colon cancer.

## Additional files

Additional file 1: **Figure S1.** A. 5-year net survivals for patients with right- and left-sided colon cancer in those aged less than 40 years old. B 5-year net survivals for patients with right- and left-sided colon cancer in those aged 40–54 years old. C 5-year net survivals for patients with right- and left-sided colon cancer in those aged 55–70 years old. D 5-year net survivals for patients with right- and left-sided colon cancer in those aged > 70 years old. (ZIP 186 kb)

Additional file 2: **Figure S2.** A. 5-year net survivals for patients with right- and left-sided colon cancer in stage localized. B 5-year net survivals for patients with right- and left-sided colon cancer in stage regional. C 5-year net survival for patients with right- and left-sided colon cancer in stage distant. (ZIP 139 kb)

Additional file 3: **Table S1.** 5-year net survival (%) and estimated excess hazard ratios for colon cancer by subsite according to age group and stage subsites, Japan, 2006–2008, for males. (DOCX 216 kb)

Additional file 4: **Table S2.** 5-year net survival (%) and estimated excess hazard ratios for colon cancer by subsite according to age group and stage subsites, Japan, 2006–2008, for females. (DOCX 218 kb)

## Abbreviations

95% CI: 95% Confidential interval
AJCC: American Joint Commission on Cancer
CRC: Colorectal cancer
DCN: Death certificate notification
DCO: Death certificate only
EHRs: Excess hazard ratios
M/I: Mortality to incidence
MCIJ: The Monitoring of Cancer Incidence in Japan
MSI: Microsatellite instability
MSS: Microsatellite stability
SEER: The Surveillance, Epidemiology, and End Results
