# Supplementary material for: Prognostic impact of tumor location in colon cancer: the Monitoring of Cancer Incidence in Japan (MCIJ) project
Source: BMC Cancer. 2019 May 9;19:431. doi: 10.1186/s12885-019-5644-y (PMC6509813; doi:10.1186/s12885-019-5644-y)
Supplement: Supplementary file 1 — Figure S1. A. 5-year net survivals for patients with right- and left-sided colon cancer in those aged less than 40 years old. B 5-year net survivals for patients with right- and left-sided colon cancer in those aged 40–54 years old. C 5-year net survivals for patients with right- and left-sided colon cancer in those aged 55–70 years old. D 5-year net survivals for patients with right- and left-sided colon cancer in those aged > 70 years old. (ZIP 186 kb) [file 12885_2019_5644_MOESM1_ESM.zip › Supplemental Figure 1DR2.docx]

Supplemental Figure 1D. 5-year net survivals for patients with right- and left-sided colon cancer in those aged > 70 years old
