# Supplementary material for: Prognostic impact of tumor location in colon cancer: the Monitoring of Cancer Incidence in Japan (MCIJ) project
Source: BMC Cancer. 2019 May 9;19:431. doi: 10.1186/s12885-019-5644-y (PMC6509813; doi:10.1186/s12885-019-5644-y)
Supplement: Supplementary file 2 — Figure S2. A. 5-year net survivals for patients with right- and left-sided colon cancer in stage localized. B 5-year net survivals for patients with right- and left-sided colon cancer in stage regional. C 5-year net survival for patients with right- and left-sided colon cancer in stage distant. (ZIP 139 kb) [file 12885_2019_5644_MOESM2_ESM.zip › Supplemental Figure 2AR2.docx]

Supplemental Figure 2A. 5-year net survivals for patients with right- and left-sided colon cancer in stage localized
