# Supplementary material for: Prognostic impact of tumor location in colon cancer: the Monitoring of Cancer Incidence in Japan (MCIJ) project
Source: BMC Cancer. 2019 May 9;19:431. doi: 10.1186/s12885-019-5644-y (PMC6509813; doi:10.1186/s12885-019-5644-y)
Supplement: Supplementary file 3 — Table S1. 5-year net survival (%) and estimated excess hazard ratios for colon cancer by subsite according to age group and stage subsites, Japan, 2006–2008, for males. (DOCX 216 kb) [file 12885_2019_5644_MOESM3_ESM.docx]

Table S1. 5-year net survival (%) and estimated excess hazard ratios for colon cancer by subsite according to age group and stage subsites, Japan, 2006-2008, for males
